# Dynamic changes in the signal-averaged electrocardiogram are associated with the long-term outcomes after ablation of ischemic ventricular tachycardia

**DOI:** 10.1007/s10840-020-00708-y

**Published:** 2020-03-02

**Authors:** Borislav Dinov, Lisa Schramm, Sebastian Koenig, Sabrina Oebel, Andreas Bollmann, Gerhard Hindricks, Arash Arya, Kerstin Bode

**Affiliations:** 1 Department of Cardiac Electrophysiology, Heart Center, University of Leipzig, Leipzig, Germany; 2 Medical Faculty, University of Leipzig, Leipzig, Germany; 3 Department of Anesthesiology, University Hospital Erlangen, Erlangen, Germany

**Keywords:** Ventricular tachycardia, Signal-averaged ECG, Catheter ablation, Late potentials, Long-term follow-up, Ischemic heart disease

## Abstract

**Purpose:**

Signal-averaged ECG (SAECG) can detect inhomogeneous myocardial conduction in patients presenting with ventricular tachycardia (VT) after myocardial infarction. Radiofrequency ablation (RFCA) aims at elimination of the endocardial late potentials and non-inducibility of VT. Previously, we demonstrated that abnormal SAECG at baseline can return to normal after a successful VT ablation. The present research investigates the post-ablation changes in SAECG after RFCA of VT and their relation to the procedural long-term outcomes.

**Methods:**

Thirty-three patients (31 male; age 68 ± 9 years; EF 36 ± 12%) with ischemic VT were prospectively enrolled to receive RFCA. One VT (range 1–7) per patient was ablated using substrate-guided RFCA and complete success was achieved in 28 (85%) cases. SAECG was performed before (t1), immediately after (t2), and at least 6 months (t3) after the RFCA.

**Results:**

After RFCA, the amount of patients showing abnormal SAECG decreased from 82% initially (t1) to 57.6% post-interventionally (t2); *P* = 0.008; and remained unchanged thereafter in 57% (t3). Patients who experienced VT recurrence (VT+) during the follow-up period had broader averaged QRS (t2): (VT+) 150 ± 26 vs. (VT−) 129 ± 21 ms; *P* = 0.015, as well as longer LAS40 (t2): (VT+) 60 ± 26 vs. (VT−) 43 ± 18 ms; *P* = 0.03. Abnormal SAECG (t2) was a strong predictor for VT recurrence: HR 5.4; 95% CI 1.5–21. SAECG detected more late potentials in patients with inferior than in those with anterior scars: 95% vs. 58%; *P* = 0.016.

**Conclusions:**

RFCA of VT in the left ventricle can improve an abnormal SAECG in some patients after myocardial infarction. Normal SAECG after RFCA of VT is associated with a lower risk for VT recurrence and death.

## Background

Survivors of myocardial infarction (MI) are at higher risk for sudden cardiac death (SCD) caused by fast ventricular tachycardia (VT) or ventricular fibrillation. The signal-averaged ECG (SAECG) is a non-invasive tool for SCD risk assessment which was extensively studied in patients with ischemic cardiomyopathy [1–3]. The time domain SAECG relies on the prolongation of the averaged QRS and the presence of low-voltage, high-frequency signals at the end of the QRS complex to identify individuals who are at risk of malignant arrhythmias. These abnormal findings are considered as a representation of inhomogeneous slow intraventricular conduction through areas of infarct scars and may predispose to occurrence of re-entry VT. During the electro-anatomical mapping abnormal fractionated and late potentials are frequently targeted as critical sites for ablation of the VT [4]. Their crucial role was demonstrated in several recent studies showing that extensive scar homogenization was associated with better outcomes than limited mapping and ablation of the clinical VT only [5, 6].

Recently, we demonstrated correlation between the size of the endocardial scar and the duration of the averaged QRS, and the late low-voltage in SAECG [7]. Additionally, we observed an improvement and even normalization of the SAECG immediately after a successful RFCA [7]. However, the dynamic changes of the SAECG recordings over time as well as their role to predict the long-term outcomes after VT ablation remain unclear. The aim of this research was to study whether the SAECG as a non-invasive tool could be useful to assess the post-interventional long-term outcomes after ablation of patients with ischemic VT. To achieve this goal, we performed SAECGs before and immediately after VT ablation. The SAECG was repeated during the long-term follow-up and the results were compared with the baseline values. We looked for association between the SAECG findings and outcomes after VT ablation and sought for prognostic factors for VT recurrence.

## Methods

### Study protocol

The study included patients after myocardial infarction and monomorphic VT who were referred for RFCA [7]. The exclusion criteria were VT of non-ischemic origin or having a reversible cause, the presence of left ventricular thrombus, and contraindications for anticoagulation with heparin, mechanical mitral or aortic valve prostheses, cardiogenic shock, and incessant VT. Initially, 50 patients were enrolled prospectively between January 2014 and May 2016; all of them received SAECG before and immediately after VT ablation. Of these, 17 cases were excluded during the course of the study. The reasons for the dropouts were as follows: a need for continuous ventricular pacemaker stimulation, CRT device implantations, and occurrence of bundle branch block [8]. Furthermore, 4 patients died and another 8 did not show up for the scheduled SAECG. Finally, 21 patients in sinus rhythm and narrow QRS complex completed the long-term follow-up after the VT ablation.

The protocol of the study complied with the Declaration of Helsinki and was approved by the institutional review board. All patients signed an informed consent for the study and for ablation.

### Signal-averaged electrocardiography

The signal-averaged ECG was performed with a commercially available GE Marquette System (Mac 5500; GE Healthcare). During the follow-up period, the SAECG was performed before the RFCA (t1), as well as immediately after RFCA (t2), and was repeated in at least 6 months after the index VT ablation (t3). Standard bipolar leads (*X*, *Y*, and *Z*) were used to acquire 250 beats at every recording. Noise levels were reduced to less than 0.5 μV. The signals were averaged and filtered with a bidirectional filter at 40–250 Hz. A vector magnitude was created by combining the three leads as follows:$\sqrt{X^2}+{Y}^2+{Z}^2$. The beginning and the end of the averaged QRS complexes were measured electronically and manually adjusted if needed. SAECG was considered positive if two out of the three parameters were abnormal: (1) filtered averaged QRS complex (fQRS) ≥ 114 ms; (2) root-mean-square voltage in the last 40 ms of the filtered QRS (RMS 40) < 20 μV; (3) duration of low-amplitude potentials < 40 μV (LAS 40) > 38 msec [9].

### Electro-anatomical mapping and catheter ablation

VT mapping and RFCA were previously described [10]. Briefly, the procedure was conducted under deep sedation with propofol. At the beginning, an attempt to induce a VT with programmed ventricular stimulation was performed using the previously described protocol [10]. All procedures were performed via transseptal approach using a steerable introducer (Agilis™, SJM, St. Paul, MN, USA). Endocardial mapping of the left ventricle was guided by an electro-anatomic mapping system (CARTO 3, Biosense Webster Inc., Diamond Bar, CA, USA), while a 7 F, 3.5-mm tip irrigated catheter (Navistar Thermocool, Biosense Webster Inc., CA, USA) was used for acquisition of the voltage points. The standard filter settings of 16–500 Hz for the bipolar Carto signals and a filling threshold of 15 were used. The scar areas were delineated using the commonly accepted thresholds for dense scar and healthy myocardium: < 0.5 and > 1.5 mV, respectively. All late and fragmented signals in the areas of low voltage were manually annotated. A temperature-controlled ablation with power settings of up to 50 W and irrigation rates of 30 ml/min was performed. The endpoint was the combination of non-inducibility of any VT with programmed ventricular stimulation and elimination of all late and fragmented potentials.

### Follow-up and study endpoint

For the purpose of the long-term follow-up, all patients were contacted via phone or mail and were scheduled for a repeated SAECG as well as an ICD interrogation at the Heart Center of Leipzig. The repeated SAECG examination was performed at least 6 months after the RFCA. Furthermore, ICD interrogations were performed every 3 months either in our Outpatient Clinic or by the referring cardiologist. The endpoint was combination of freedom from any VT requiring ATP, ICD shock, or cardiac death.

### Definitions of the clinical covariates

1. Abnormal SAECG—SAECG with at least two abnormal characteristics: averages QRS ≥ 114 ms; LAS40 > 38 ms, RMS 40 < 20 μV
2. Scar—the endocardial surface area exhibiting low-voltage electrograms with amplitude < 1.5 mV.
3. Acute procedural success—elimination of all inducible VT; on the other hand, procedural failure—persistent re-induction and failure to eliminate all inducible monomorphic Vts
4. Low ejection fraction measured by echocardiography using the biplane Simpson’s method < 35%

### Statistical analysis

Continuous variables with normal distribution were presented as mean value ± SD; otherwise, median with range was given. Categorical variables were reported as absolute numbers and percentages. To compare differences between groups, Student *t* test, chi-square, and Fischer’s exact test were used. Comparison of SAECG at different examination points was performed using the paired *t* test (regarding characteristics LAS 40, RMS 40 and fQRS), and McNemar and Wilcoxon signed-ranks tests (regarding the proportions of abnormal SAECG tests) for patients with and without VT recurrence. Event-free survival was estimated with Kaplan-Meier method. We used Cox proportional hazards model with backward conditional elimination to find the predictors for the combined endpoint of VT recurrence/death. As dependent variable, we used the time to first VT recurrence or death. We decided to include in the model potentially relevant variables as well as variables that showed significant difference between the VT recurrence/no recurrence groups in the univariate analysis with a two-sided *P* < 0.1. A statistical significance was considered as two-sided *P* < 0.05. All analyses were performed with SPSS 20.0 (IBM, Armonk, NY, USA).

## Results

### Patient characteristics

Thirty-three patients (31 male; mean age 67.5 ± 9 years) were included in the study. The mean LV EF was 36 ± 12% and the LV EDV 190 ± 50 ml. Fourteen patients (42.5%) had severe heart failure NYHA class ≥ 3. Twenty-one had an ICD at admission and all of them had an ICD at discharge from the hospital. The indications for RFCA were electrical storm in 11 patients and recurrent sustained VTs in the rest. The baseline clinical characteristics are summarized in Table 1.

**Table 1:** Baseline clinical characteristics

Baseline characteristics (*N* = 33)	Value
Age, years	67 ± 9
Male gender, *n* (%)	31 (93.9)
Arterial hypertension, *n* (%)	27 (81.8)
Diabetes mellitus, *n* (%)	7 (21.2)
Coronary artery disease	
1-Vessel, *n* (%)	15 (45.5)
2-Vessel, *n* (%)	11 (33.3)
3-Vessel, *n* (%)	7 (21.2)
Coronary artery bypass, *n* (%)	7 (21.2)
Anterior scar/inferior scar, *n*	12/21 (100)
Electrical storm, *n* (%)	11 (33.3)
Atrial fibrillation/flutter, *n* (%)	13 (39.4)
Heart failure NYHA III and IV*	14 (42.5)
LV ejection fraction, %	36 ± 12
LV Enddiastolic volume, ml	190 ± 50
ICD at discharge, *n* (%)^¶^	33 (100)
Beta-blocker, *n* (%)	30 (91)

*New York Heart Association (classification of heart failure)

^¶^Implantable cardioverter defibrillator

### VT ablation procedure and long-term outcomes

RFCA resulted in complete elimination of all inducible VTs in 28 (85%) patients. At least one VT (range 1–7) per patient was ablated. The mean procedure time was 135 ± 51 min and the mean RF time was 1055 ± 572 s. The mean scar area (0.5–1.5 mV) was 35 ± 21 cm^2^; the mean ratio of scar area to LV surface was 20 ± 21%. The mean dense scar area (< 0.5 mV) was 12 ± 13 cm^2^ and the dense scar area to LV area ratio was 6.5 ± 6.8% (Table 2). No major procedure-related complications occurred. During the follow-up period of mean 17 ± 4 months, VT recurrence was observed in 13 cases and death occurred in 4 patients.

**Table 2:** Mean procedural values of RFCA

	Mean values
Procedure time (min)	135 ± 51
Radiofrequency time (sec)	1055 ± 572
Scar area 0.5–1.5 mV (cm^2^)	35 ± 21
Ratio of scar area to LV surface (%)	20 ± 21
Dense scar area < 0.5 mV (cm^2^)	12 ± 13
Dense scar area to LV area ratio (%)	6.5 ± 6.8

*RFCA*, radiofrequency catheter ablation

### Differences in SAECG in relation to the scar localization

Of 33 patients, 21 had inferior located low-voltage areas and the remaining 12 had scars with apical, apico-septal, or anterior localization. Patients with inferior MI had better LV EF than those with anterior MI: 42 ± 10% vs. 26 ± 8%; *P* = 0.0001. Also, a trend towards less extensive low-voltage areas (< 1.5 mV) in patients with inferior MI was observed: 30 ± 19 cm^2^ vs. 44 ± 21 cm^2^; *P* = 0.06 and dense scar < 0.5 mV: 10 ± 12 cm^2^ vs. 16 ± 16 cm^2^; *P* = 0.25. There was no difference in the number of inducible VTs between patients with inferior and anterior infarction: №VT 2 ± 1.7 vs. №VT 2.6 ± 2.1; *P* = 0.35, respectively.

Before RFCA, SAECG (t1) was abnormal in 20 out of 21 (95%) cases with inferior scars and in 7 out of 12 (58%) in those with anterior scars; *P* = 0.016. The duration of the averaged fQRS was similar in patients with inferior and anterior scars: fQRS inf. 139 ± 22 ms vs. fQRS ant. 133 ± 19 ms; *P* = 0.449. However, in those with inferior MI, the duration of LAS40 was longer 57 ± 22 ms vs. 40.5 ± 18 ms; *P* = 0.033, and the RMS40 amplitude was lower 13 ± 10 μV vs. 27 ± 19 μV; *P* = 0.01. Results are presented in Table 3.

**Table 3:** Differences in SAECG in relation to the scar localization

Variables	Inferior MI	Anterior MI*	*P*
Patients (*n*)	21	12	
LVEF (%)^¶^	42 ± 10	26 ± 8	0.0001
Low-voltage area < 1.5 mV (cm^2^)	30 ± 19	44 ± 21	0.06
Dense scar < 0.5 mV (cm^2^)	10 ± 12	16 ± 16	0.25
Inducible VT (*n*)	2 ± 1.7	2.6 ± 2.1	0.35
SAECG (t1) abnormal (%)	95	58	0.016
fQRS (ms)	139 ± 22	133 ± 19	0.449
LAS40 (ms)	57 ± 22	40.5 ± 18	0.033
RMS40 (μV)	13 ± 10	27 ± 19	0.01

*Myocardial infarction with an apical, apico-septal, or anterior localization

^¶^Left ventricular ejection fraction

### Dynamic changes in SAECG during long-term follow-up

**Fig. 1:**
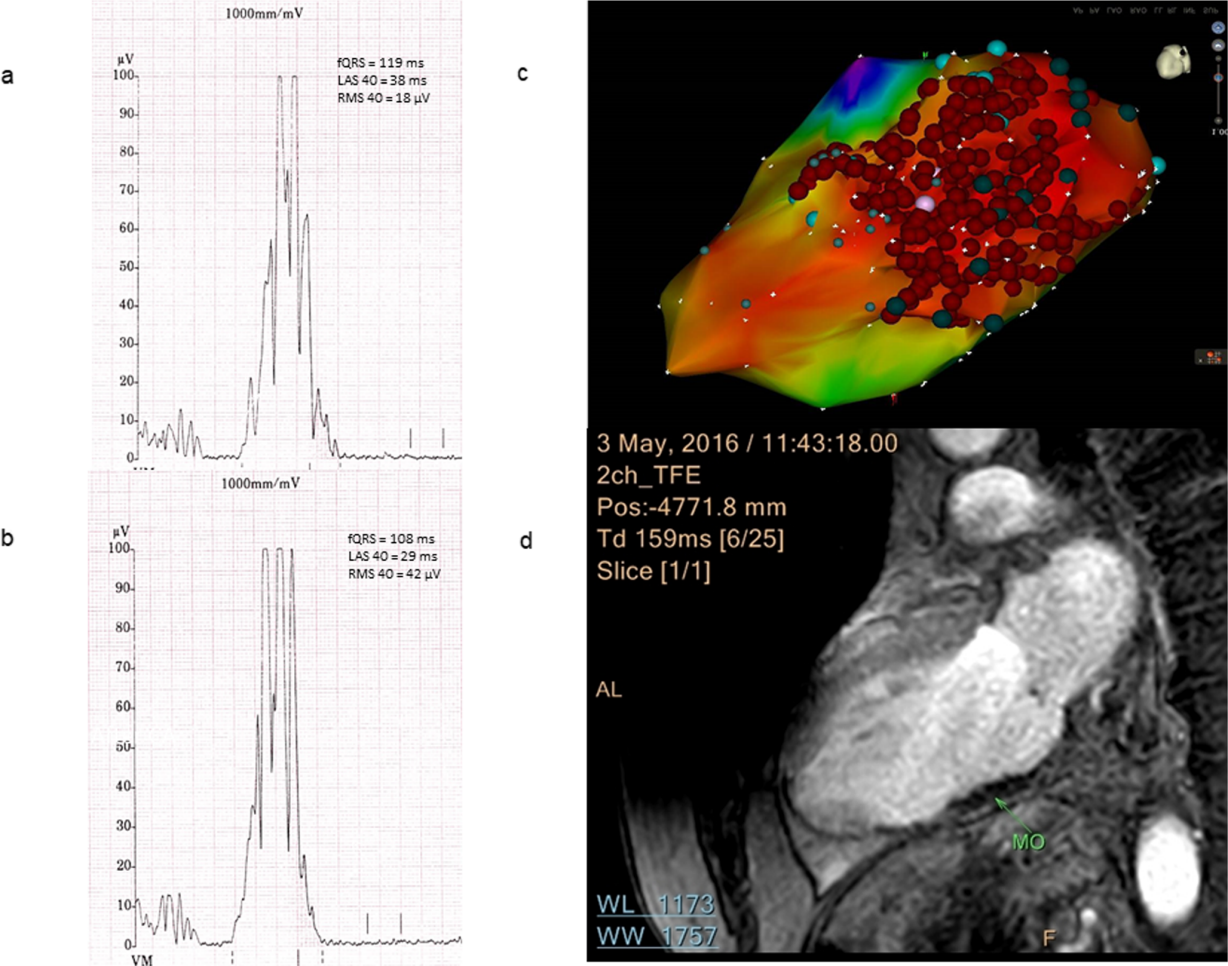
Example of an improvement in SAECG. An example of SAECG before ablation (**a**) which improved afterwards (**b**) and the voltage map with the ablation points (**c**). The corresponding CMR-LGE (cardiovascular magnetic resonance imaging with late gadolinium enhancement (**d**)) shows an area of microvascular obstruction due to CA

**Fig. 2:**
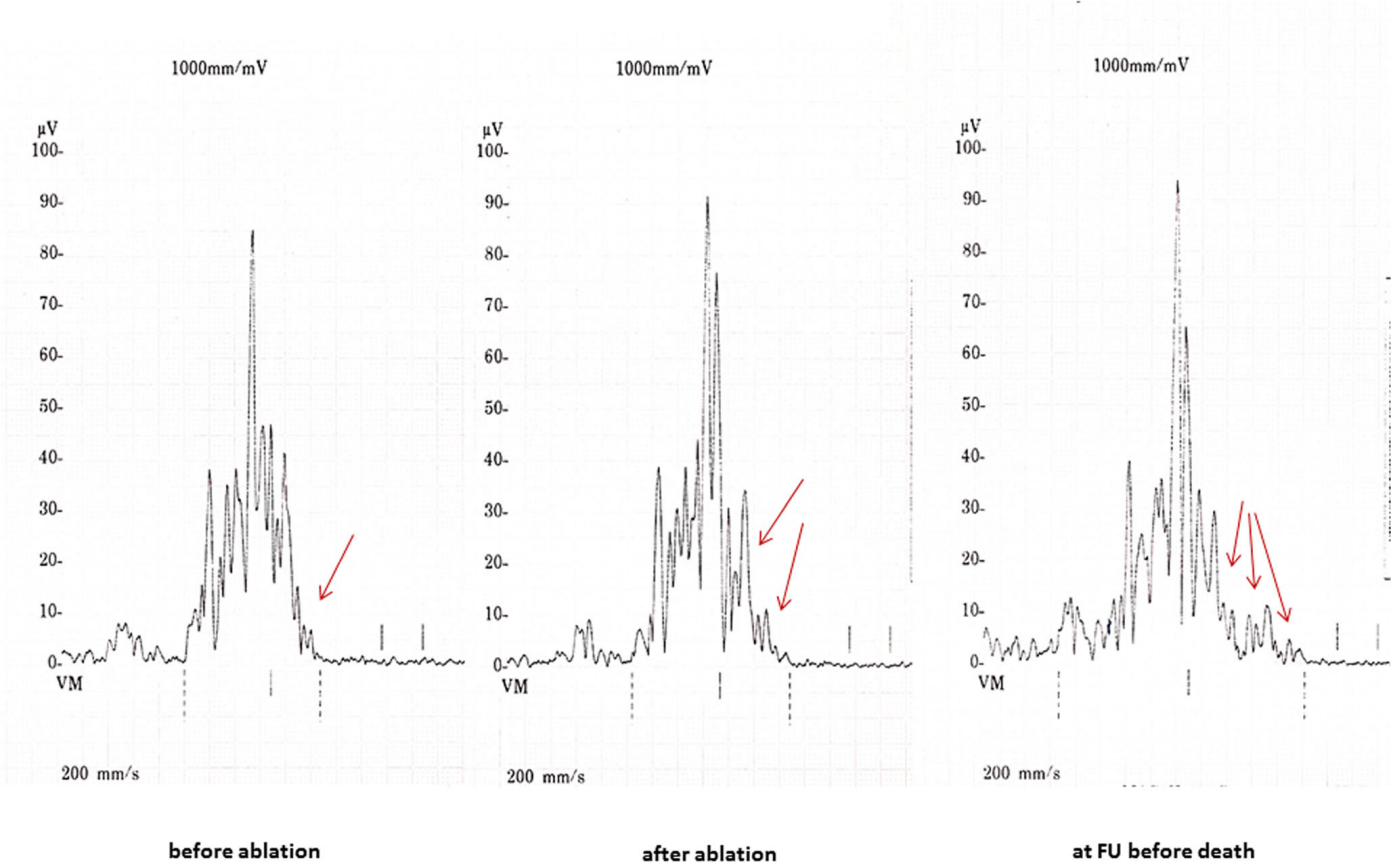
Example of a SAECG that worsened during the follow-up period

**Fig. 3:**
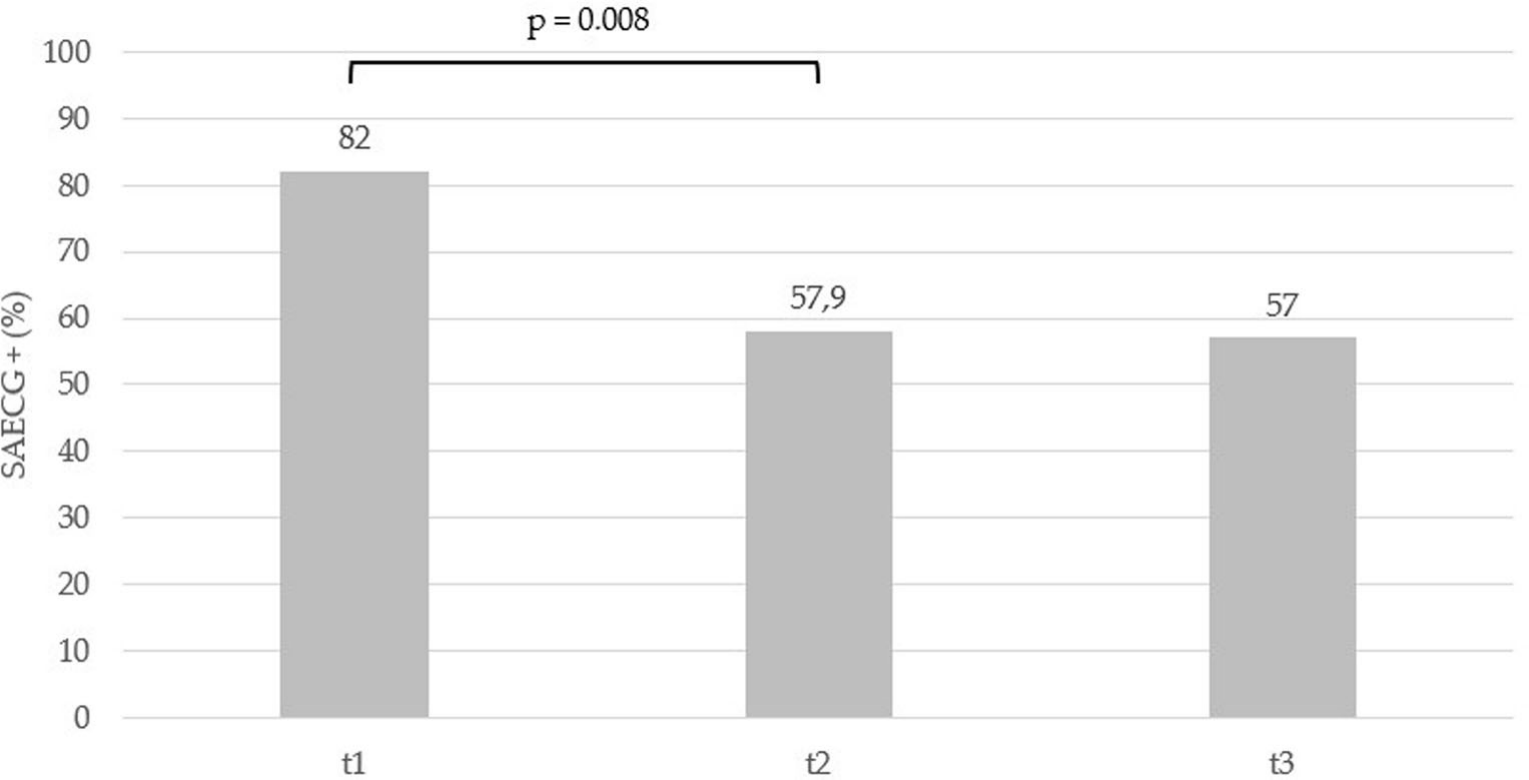
Changes of the SAECG before (t1), immediately after VT ablation (t2), and at long-term follow-up (t3). The bars represent the proportion of patients which had abnormal SAECG before (t1), after VT ablation (t2), and at follow-up (t3). A significant decrease in the proportion of the abnormal SAECG after RFCA in comparison with baseline was observed

Before VT ablation, SAECG (t1) was pathological in 27 (82%) out of 33 patients. In the VT recurrence group (13 patients), 12 (92%) patients had abnormal SAECG (t1) while in the non-recurrence group (20 patients), 15 (75%) patients demonstrated abnormal SAECG (t1); *P* = 0.364. Post-interventionally, SAECG (t2) returned to normal in 8 patients, resulting in significantly less abnormal SAECG after RFCA (t2) compared with SAECG before RFCA (t1): 19 (57.6%) vs. 27 (82%) patients respectively; *P* = 0.008. Examples of SAECGs after RFCA are shown in Figs. 1 and 2. At the end of the follow-up (t3), SAECG (t3) was available in 21 patients. Of these, the SAECG (t3) remained abnormal in 12 (57%) cases, which is a comparable finding with the SAECG immediately after RFCA (t2) (Fig. 3).

Before RFCA, the duration of the averaged fQRS (t1) was longer in the group with VT recurrence (VT+) than in the group without VT recurrence (VT−): fQRS (t1) (VT+) = 148 ± 24 ms vs. fQRS (t1) (VT−) = 130 ± 16 ms; *P* = 0.02. Similar finding was observed for LAS40 (t1) before RFCA: LAS40 (t1) (VT+) = 60 ± 26 ms vs. LAS40 (t1) (VT−) = 45.5 ± 17 ms; *P* = 0.065. Immediately after RFCA (t2), the averaged QRS in the VT recurrence group remained significantly longer: fQRS (t2) (VT+) = 150 ± 26 ms vs. fQRS (t2) (VT−) = 129 ± 21 ms; *P* = 0.015. Notably, a shortening of the LAS 40 (t2) was observed after RFCA in the group without VT recurrence: LAS40 (t2) (VT−) = 42.5 ± 18 ms vs. LAS40 (t2) (VT+) = 60 ± 26.5 ms; *P* = 0.03.

**Fig. 4:**
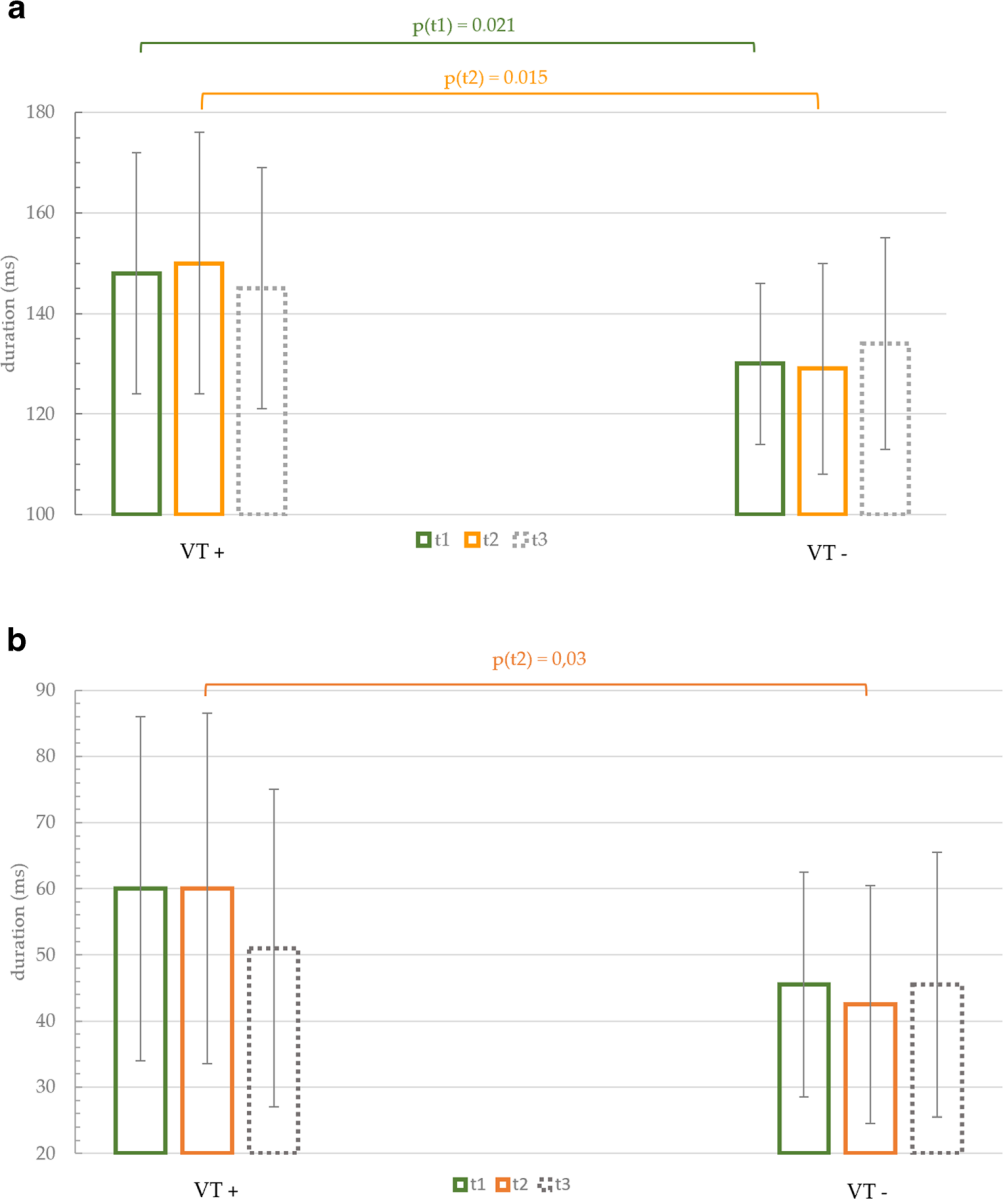
Temporal changes in the duration of the QRS and LAS40 in patients with and without VT recurrence during the follow-up. **a** The bars illustrating the differences between the fQRS in patients with and without VT recurrence before, immediately after, and at long-term FU after RFCA. Patients with VT recurrence had significantly wider fQRS before and after RFCA. **b** The bars representing the differences in duration of LAS40 in patients with and without VT recurrence. LAS 40 after RFCA (t2) was significantly longer in patients with VT recurrence than in those without VT recurrence

At long-term follow-up, fQRS (t3) (VT+) was 145 ± 24 ms, while fQRS (t3) (VT−) became longer: 134 ± 21 ms; *P* = 0.28. No changes in LAS 40 (t3) were observed between the groups with and without VT recurrence: LAS 40 (t3) (VT+) = 51 ± 24 ms vs. LAS40 (t3) (VT−) = 45.5 ± 20 ms; *P* = 0.49. Regarding RMS, no significant changes between patients with and without VT recurrence were observed before, immediately after, and at long-term follow-up after VT ablation. Significant values are presented in Fig. 4.

### Long-term changes in SAECG in patients with VT recurrence

In the subgroup of patients with VT recurrence, a shortening of LAS40 was observed at the end of the follow-up period compared with the baseline: LAS 40 (t3) = 51 ± 24 ms vs. LAS40 (t1) = 65 ± 31 ms; *P* = 0.033. On the other hand, the duration of the fQRS (t3) and the amplitude of RMS 40 (t3) did not change in comparison with the baseline values in SAECG (t1).

### Predictors for VT recurrence and death

**Fig. 5:**
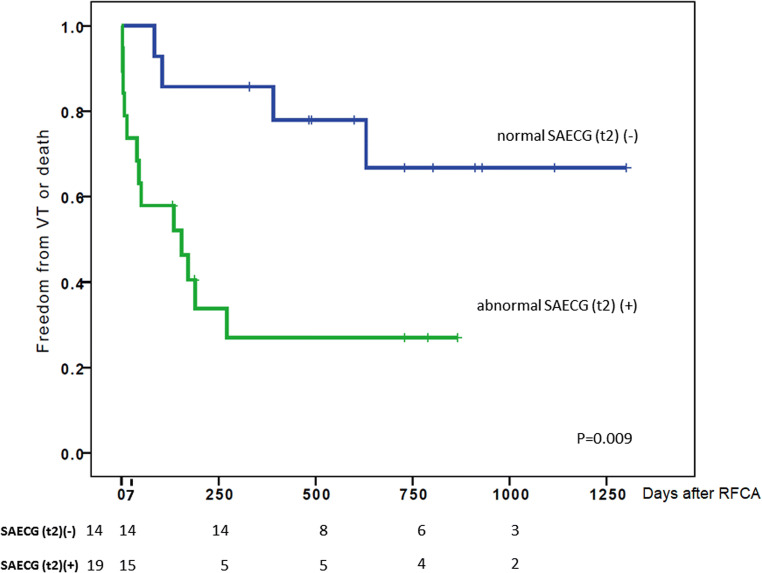
Freedom from VT recurrence and death in patients with normal and abnormal SAECG after RFCA. The Kaplan-Meyer curves for the combined endpoint freedom from VT recurrence and death. Patients with abnormal SAECG after RFCA (t2) had a higher likelihood for VT recurrence and death than patients with normal SAECG after RFCA (t2)

The Kaplan-Meyer analysis showed that the presence of abnormal SAECG after RFCA (t2) is associated with a higher probability of combined endpoint of VT recurrence and death (*P* = 0.009). The Kaplan-Meyer curves for the freedom from VT recurrence and death are presented in Fig. 5.

In the univariate analysis, the presence of abnormal SAECG after ablation (t2), as well as electrical storm before admission, lower EF and failure to achieve successful ablation of the VT were predictors for VT recurrence and death, while scar area and age were not. In the multivariate Cox proportionate analysis, the following co-variables were included: abnormal SAECG after ablation, electrical storm at admission, EF, age, left ventricular scar area, and incomplete ablation. A backward elimination approach was used and the final model contained only 2 variables as significant predictors for VT recurrence: abnormal SAECG (t2) (HR 5.69; CI 95% 1.54–20.9; *P* = 0.009), and incomplete ablation (HR 8.33; CI 95% 1.76–39.5; *P* = 0.008). The results are presented in Table 4.

**Table 4:** Predictors for VTrecurrence and death after RFCA^#^

Variables	HR Univariate analysis	95% CI	*P*	HR Multivariate analysis	95% CI	*P*
Age	0.98	0.94–1.04	0.676	0.98	0.92–1.04	
Electrical storm	25.5	3.52–184	0.001	1.19	0.36–3.94	
LVEF, %	0.94	0.89–0.99	0.009	0.95	0.89–1.01	
SAECG (t2)*	4.19	1.34–13.1	0.013	5.69	1.54–20.9	0.009
Scar area, cm^2^	1.02	0.99–1.04	0.100	1.02	0.99–1.05	
Incomplete RFCA	3.89	1.25–12.3	0.021	8.33	1.76–39.5	0.008

^#^Cox regression model with backward conditional elimination

*SAECG (t2), abnormal SAECG after RFCA

*RFCA*, radiofrequency catheter ablation

## Discussion

SAECG is a non-invasive tool for evaluation of the risk for malignant VA in patients after MI whose clinical significance declined with the widespread use of decreased LVEF for identifying patients at risk for SCD. However, the abnormal late potentials in the SAECG are considered to represent a substrate for reentrant VT and have a good positive predictive value for occurrence of malignant VA. In several studies, the presence of abnormal SAECG in patients with non-sustained VT was associated with higher likelihood for induction of VT using programmed electrical stimulation [11–13]. Recently, a novel method called layered symbolic decomposition (LSDf) that quantifies the percentage of hidden QRS wave frequency components in SAECG was evaluated as a non-invasive tool for SCD risk stratification. In a study, patients who received ICD therapy for VT/VF had significantly lower LSDf compared with arrhythmia-free patients [14]. In the context of VT ablation, in a previous smaller study of 4 patients with ischemic VT, the catheter ablation that targeted only the middiastolic potentials during ongoing VT failed to improve the SACEG [15]. Since then, the VT ablation technique shifted from activation mapping towards more effective, extensive substrate modification aiming at the elimination of all abnormal electrograms [5, 6, 16, 17]. Recently, we demonstrated that extensive substrate-guided ablation resulted in acute normalization of the SAECG in some patients after ablation [7]. Similar observation was made by Breithardt et al. who showed that late potentials in SAECG of patients with post-MI ventricular tachycardia disappeared acutely after successful surgical resection of the LV scar [18]. However, there is limited data regarding the dynamic changes in the SAECG after VT ablation over a longer follow-up period. In the present study, we aimed to explore the changes in the SAECG in relation to the long-term outcomes after RFCA.

In the present cohort of 33 survivors of MI who were referred for ablation of sustained VT, we observed a post-interventional normalization of the SAECG in 8 cases. Furthermore, patients with normal SAECG after RFCA had significantly less VT recurrences than those with abnormal SAECG. In a study with a similar design, Liao et al. described an improvement of the SAECG after RFCA in patients with ARVC in a follow-up after 3 months, which was also associated with a lower probability for VT recurrence [19]. Additionally, in our study, the inducibility of VT and the presence of abnormal SAECG after RFCA were stronger predictors for VT recurrence than the LV EF. In line with our study, the group around Pappone observed similar associations between the SAECG recordings and the severity of the phenotype in patients with Brugada syndrome. Recently, they found that the duration of the averaged QRS complex and the LAS was significantly longer in patients with a spontaneous type 1 Brugada ECG. Moreover, the size of the epicardial substrate during RF ablation was predictor for abnormal SAECG in patients with Brugada syndrome [20].

The improvement of the SAECG after RFCA was mainly caused by shortening of the fQRS and the LAS40, without significant changes in the RMS40 amplitude. Patients who had experienced VT recurrence during the follow-up demonstrated significantly longer fQRS and LAS40 after RFCA. These findings are further supported by our previous observations that fQRS and LAS40 showed a positive correlation with the size of the endocardial low-voltage areas [7]. All these observations suggest that the subtle changes of the SAECG can be used as an adjunctive tool to evaluate the efficacy of the RFCA. An additional evidence for this was provided by Dailey et al. who demonstrated that intracoronary infusion of ethanol can eliminate the late potentials in SAECG and terminate the VT [21]. Notably, RMS40 was not affected by the RFCA and did not show any difference between patients with and without VT recurrence, suggesting rather low sensitivity to detect modifications of the arrhythmogenic substrate and therefore can possibly be omitted as a component of the SAECG.

In this study, we observed significant differences in the SAECG recordings depending on the site of the myocardial infarction. Almost all patients with inferior scars demonstrated abnormal SAECG, while only half of the cases with anterior scars exhibited pathological SAECG recordings. In addition, the averaged QRS and LAS40 were significantly longer in case of inferior MI. The finding of more abnormal SAECG in patients with inferior MI was in contrast with the better LV EF and the smaller LV scars in these patients. Previously, Gomes et al. also observed higher prevalence of abnormal SAECG in patients with inferior MI compared with those with anterior myocardial infarction [22]. A plausible explanation is that the infero-posterior part of the left ventricle is the last to be depolarized and leads to lagging electrical activation of inferior scars. Certain selection bias may also play role, because of the higher prevalence of left bundle branch block in patients with anterior MI, which is normally an exclusion criterion in most studies with SAECG, including ours. Therefore, the changes of the SAECG after RFCA must be carefully interpreted, having in mind the localization of the myocardial scar. In this study, the SAECG after 6 months did not show any further fluctuations except from the acute changes after RFCA. However, if a VT ablation needs to be repeated due to VT recurrences, it is reasonable to obtain a new SAECG before and after the procedure in order to assess the procedural success.

The results of our research suggest that the usefulness of the SAECG can reach beyond its role as a method for SCD prediction and can be used as an inexpensive and low-risk tool for assessment of the procedural success. Larger prospective studies are needed to evaluate its predictive value and limitations in different clinical settings and patient cohorts.

## Study limitations

This investigation serves as a pilot study. The small number of patients and the relatively short follow-up period are limitations. The effect of the anti-arrhythmic therapy on the SAECG recordings was not evaluated; however, the antiarrhythmic drugs before RFCA were maintained after the intervention in all but two patients who discontinued their therapy with amiodarone and remain free of any VT thereafter.

## Conclusions

Abnormal SAECG can improve to normal in some patients with MI after VT ablation, although, in most of them, the SAECG recordings were unaffected by the VT ablation and remained constant over time. Significant changes were observed in LAS40 and the duration of the averaged QRS but not in the RMS40. Patients in whom the SAECG changed to normal after RFCA had lower risk for VT recurrence. The sensitivity of the SAECG might be lower in patients with anterior MI.
