# Correction to: Dynamic changes in the signal-averaged electrocardiogram are associated with the long-term outcomes after ablation of ischemic ventricular tachycardia

**DOI:** 10.1007/s10840-021-01038-3

**Published:** 2021-07-31

**Authors:** Borislav Dinov, Lisa Schramm, Sebastian Koenig, Sabrina Oebel, Andreas Bollmann, Gerhard Hindricks, Arash Arya, Kerstin Bode

**Affiliations:** 1 Department of Cardiac Electrophysiology, Heart Center, University of Leipzig, Leipzig, Germany; 2 Medical Faculty, University of Leipzig, Leipzig, Germany; 3 Department of Anesthesiology, University Hospital Erlangen, Erlangen, Germany

**Correction to: Journal of Interventional Cardiac Electrophysiology (2021) 60:125–134**

**https://doi.org/10.1007/s10840-020-00708-y**

The article “Dynamic changes in the signal-averaged electrocardiogram are associated with the long-term outcomes after ablation of ischemic ventricular tachycardia”, written by Borislav Dinov, Lisa Schramm, Sebastian Koenig, Sabrina Oebel, Andreas Bollmann, Gerhard Hindricks, Arash Arya and Kerstin Bode was originally published Online First without Open Access. After publication in volume 60, issue 1, page 125–134 the author decided to opt for Open Choice and to make the article an Open Access publication. Therefore, the copyright of the article has been changed to © The Author(s) 2020 and the article is forthwith distributed under the terms of the Creative Commons Attribution 4.0 International License, which permits use, sharing, adaptation, distribution and reproduction in any medium or format, as long as you give appropriate credit to the original author(s) and the source, provide a link to the Creative Commons licence, and indicate if changes were made. The images or other third party material in this article are included in the article’s Creative Commons licence, unless indicated otherwise in a credit line to the material. If material is not included in the article’s Creative Commons licence and your intended use is not permitted by statutory regulation or exceeds the permitted use, you will need to obtain permission directly from the copyright holder. To view a copy of this licence, visit http://creativecommons.org/licenses/by/4.0.

The original article has been corrected.

